# Prevalence of pica and rumination behaviours in adults and associations with eating disorder and general psychopathology: findings form a population-based study

**DOI:** 10.1017/S2045796022000208

**Published:** 2022-06-09

**Authors:** A. S. Hartmann, M. Zenger, H. Glaesmer, B. Strauß, E. Brähler, M. de Zwaan, A. Hilbert

**Affiliations:** 1 Department of Psychology, University of Konstanz, Konstanz, Germany; 2 Faculty of Applied Human Studies, University of Applied Sciences Magdeburg and Stendal, Stendal, Germany; 3 Department of Psychosomatic Medicine and Psychotherapy, University of Leipzig Medical Center, Integrated Research and Treatment Center AdiposityDiseases, Behavioral Medicine Research Unit, Leipzig, Germany; 4 Department of Medical Psychology and Medical Sociology, University of Leipzig, Leipzig, Germany; 5 Universitätsklinikum Jena, Friedrich-Schiller-Universität, Institut für Psychosoziale Medizin, Psychotherapie und Psychoonkologie, Jena, Germany; 6 Department of Psychosomatic Medicine and Psychotherapy, University Medical Center Mainz, Mainz, Germany; 7 Department of Psychosomatic Medicine and Psychotherapy, Hannover Medical School, Hannover, Germany

**Keywords:** Adults, pica, population-based study, prevalence, rumination disorder

## Abstract

**Aims:**

Pica and rumination disorder are known as feeding disorder diagnoses in childhood, but little is known about their occurrence in adulthood. This study aimed to assess prevalence rates of one-time and recurrent pica and rumination behaviours (PB and RB) in adults, including sociodemographic subgroups, and to examine associations with other eating disorder and general psychopathology.

**Methods:**

The representative population sample (*N* = 2403) completed measures on PB and RB, symptoms of avoidant/restrictive food intake disorder (ARFID), body image and symptoms of depression and anxiety.

**Results:**

Any PB and RB were reported in 5.33 and 5.49%, respectively, while recurrent PB or RB occurred in 1.08 and 0.71%, respectively. Co-occurrence was high, with 35.29% of recurrent PB in RB, and 23.08% vice versa. Prevalence rates of recurrent PB or RB did not differ by gender, weight status, educational or migration history from those without recurrent behaviours. Adults with *v*. without recurrent PB and RB showed more symptoms of ARFID, general eating disorders depression and anxiety, and behavioural symptoms of eating disorders (with the exception of compensatory behaviours in recurrent PB), and less positive body image. However, there were no differences regarding age and body mass index.

**Conclusions:**

Our findings highlight the clinical significance of PB and RB in adults regarding both prevalence and associations with other psychopathological symptoms. In particular, associations with body image need to be investigated further, as in contrast to other eating disorders, body image disturbance is not yet represented in the diagnostic criteria for pica and rumination disorder. In sum, the findings highlight the need for clinical attention for these disorders and related behaviours in adults.

## Introduction

Pica and rumination disorder are two new diagnostic entities in the fifth edition of the Diagnostic and Statistical Manual of Mental Disorders (DSM-5; American Psychiatric Association, APA, 2013) and the 11th version of the International Classification of Diseases (ICD-11; World Health Organization, WHO, 2021). Compared to the former classification in the category Mental Disorders with Onset in Childhood or Adolescence in the DSM-IV (APA, 1996) and the ICD-10 (WHO, 1993), this new prominent placement in the category Feeding and Eating Disorders (DSM-5) and in the category Feeding or Eating Disorders (ICD-11) highlights their potential lifelong relevance. Both disorders can be physically detrimental (e.g. obstructions or perforations of the gastrointestinal tract in pica, and malnourishment in rumination disorder; Decker, 1993; Chial *et al*., 2003; Luoba *et al*., 2005; Stiegler, 2005). Against this background, it is surprising that their prevalence in the general population, comorbid symptom presentation and relations to other eating disorder symptoms in adults have barely been studied.

Pica is defined as the consumption of substances that are neither edible nor nutritious (APA, 2013). Potential substances vary greatly, for instance sand, paper or faeces (Leung and Hon, 2019), and craving for the substance can be strong (Young, 2011; Sturmey and Williams, 2016). It is only diagnosed if symptoms have been present for longer than one month and patients show a developmental age of at least 2 years. Pica behaviour (PB) must not be socially or culturally accepted, and should be severe enough to warrant clinical attention as pica disorder beyond the treatment of comorbid diagnoses (APA, 2013).

Rumination disorder is characterised by recurrent regurgitation of previously ingested food. The brought-up food is subsequently re-chewed, re-swallowed or spit out. Regurgitation usually seems effortless and is not accompanied by nausea (Stanghellini *et al*., 2016). Moreover, the food is only minimally digested and thus still has a pleasant taste. Symptoms must be present over at least one month and occur repeatedly over one year (APA, 2013). Diagnosis can only be given if the behaviour is not due to a physical condition and does not only occur in the course of another eating disorder. As with PB, rumination behaviour (RB) needs to be severe enough to warrant clinical attention as rumination disorder beyond comorbid diagnoses (APA, 2013).

Despite the definition of diagnostic criteria for both disorders across the lifespan, epidemiological data on the full-syndrome disorders or PB and RB in the general population are scarce. According to the DSM-5, the prevalence of pica is unclear (APA, 2013), and epidemiological studies in the general adult population are lacking. A study in *N* = 100 adult females and males with overweight or obesity at a weight-loss clinic indicated frequent PB (Delaney *et al*., 2015). Moreover, a literature review of six studies suggested a high PB prevalence in individuals with intellectual disabilities both in community settings (from 0.3 to 14.4%) and in institutionalised populations (from 9 to 25%; Ali, 2001). Pregnant women have also been proposed as a high-prevalence population: A meta-analysis integrating 70 studies yielded an aggregate PB prevalence estimate of 27.8% (95% confidence interval (CI) 22.8–33.3; Fawcett *et al*., 2016), although with strong geographical differences, for example, pregnant European women hardly exhibited PB. Pica prevalence in this meta-analysis was also greater in individuals with lower education and higher anaemia (Fawcett *et al*., 2016). Notably, both reviews only included studies conducted prior to the introduction of the DSM-5 (APA, 2013), and assessment methods and diagnostic/definition criteria varied vastly. To our knowledge, only one population-based study has assessed PB, indicating at least one-time PB in 12.31% and recurrent PB in 4.98% of youth aged from 7 to 14 years (Hartmann *et al*., 2018).

Epidemiological studies assessing rumination disorder or RB in the general adult population are lacking. A narrative review article indicated that the only existing data from clinical practice are probably inadequate for epidemiological analyses regarding this underdiagnosed condition, which is often overlooked in the differential diagnosis of refractory vomiting or regurgitation (Tack *et al*., 2011). The authors concluded that given the case load at referral centres, rumination disorder is presumably uncommon in adults (Tack *et al*., 2011). The aforementioned study by Delaney *et al*. indicated frequent RB in adult individuals with overweight and obesity (Delaney *et al*., 2015). The only population-based study in youth regarding RB yielded prevalences of 11.94% of any RB and 1.49% of recurrent RB (Hartmann *et al*., 2018).

Not only is there a lack of knowledge regarding prevalences of the full-syndrome disorders or of PB and RB, but it is also widely understudied how significant the associated psychological burden (i.e. comorbid psychopathology) is across the age range. PB has been associated with intellectual disabilities (Sturmey and Williams, 2016) and with symptoms of obsessive-compulsive disorder (Bharti *et al*., 2015) and autism spectrum disorder (Clark *et al*., 2010). Reported comorbid conditions in RB across different age groups are functional gastrointestinal disorders (Rajindrajith *et al*., 2012), disorders with vomiting (Murray *et al*., 2019), stomach aches and weight loss (Chial *et al*., 2003; Rajindrajith *et al*., 2012). While Cai *et al*. (2021) found that mental disorders and eating disorders are independent risk factors for rumination disorder, to the best of our knowledge, there are no systematic investigations assessing associations of PB and RB with general psychopathology in adults. The aforementioned study in 7 to 14-year-olds found no associations of PB and RB with general psychopathology but reported small correlations with symptoms of avoidant/restrictive food intake disorder (ARFID) (Hartmann *et al*., 2018), with the latter finding being confirmed in another primary school-based sample of children (Murray *et al*., 2018). This association with the psychopathology and behavioural symptoms of eating disorders is in line with the aforementioned study in adolescents and young adults in residential eating disorder care, which reported prevalences of 1.3 and 7.4% for PB and RB, respectively (Delaney *et al*., 2015).

Against this background, the present study aimed to assess the prevalences of PB and RB using self-report measures in the adult population and in specific demographic groups. A second aim was to measure associated eating disorder and general psychopathology. We expected prevalences to be lower than those shown in a population-based study in children (Hartmann *et al*., 2018), given that the onset of PB and RB is for the greater part reported in childhood (DSM-5; APA, 2013) and spontaneous remissions might occur over time. From an exploratory perspective, we examined 10-year age brackets of our sample in terms of the prevalences of both disorders. From a sociodemographic perspective, in line with previous research, we hypothesised that men would report greater prevalences of recurrent PB than women; that individuals with underweight, overweight and obesity would report both recurrent PB and RB more often than those with normal weight (Delaney *et al*., 2015); and that individuals with a lower educational level would report a higher prevalence of recurrent PB. Potential variations of recurrent RB by gender and education were addressed from an exploratory perspective. Regarding associations with psychopathology, we assumed greater eating disorder psychopathology, more behavioural symptoms of eating disorders (i.e. binge eating and compensatory behaviours), more symptoms of ARFID, less positive body image and higher depressive and anxiety symptoms in adults with *v*. without recurrent PB or RB.

## Methods

### Recruitment and sample

Recruitment procedures have been extensively described by Hilbert *et al*. (2021). Participants were required to be ⩾ 14 years old (for the overall project; the present paper focuses on adults (⩾18 years old) only) and fluent in German. During September to November 2016, we randomly recruited a representative sample of German individuals with the support of the independent agency of market, opinion and social research USUMA (Berlin, Germany). The three stages of the sampling procedure included sample point regions from 258 regions in Germany, target households within these sample point regions based on a random route procedure and target persons within the selected households through a Kish selection grid, based on a pre-assigned table for selection of target persons. Out of 4902 target households, *N* = 2510 individuals agreed to participate (response rate 51.20%). Of those not participating (*n* = 2392), *n* = 738 households refused, 723 households were not reachable, nine households were unoccupied, in 15 households no individual met inclusion criteria, 715 target persons refused, 111 target persons were not reachable (after a maximum of four attempts) and of 81 target households/persons reasons for non-participation are unknown. As the study aimed to examine PB and RB in adults, we excluded participants aged <18 years (*n* = 86), yielding a sample of *N* = 2424. Of these participants, data on PB and/or RB were missing for *n* = 21, resulting in a final sample of *N* = 2403.

### Procedure

A trained research assistant met participants at their home, informed them about the study procedure and obtained informed consent. The research assistant then asked participants to complete self-report assessments. The Ethics Committee of the University of Leipzig approved the study protocol (No. 297/16-ek). The study followed the ethical guidelines of the International Code of Marketing and Social Research Practice by the International Chamber of Commerce and the European Society for Opinion and Marketing Research.

### Measures

*Demographic and anthropometric data*: Participants provided information regarding their age, gender (the German term ‘Geschlecht’ covers both gender and sex; participants were asked ‘Which gender [Geschlecht] do you belong to [male, female, N/A]?’), nationality (German *v*. other) and highest educational attainment (which was subsequently dichotomised into <12 *v*. ⩾12 years, with 12 years of education reflecting the minimum school duration needed to obtain a university entry-level diploma). Moreover, they reported their current weight and height, which we used to calculate body mass index (BMI) as kg/m^2^. Based on their BMI, participants were placed in weight categories (underweight: BMI < 18.5, normal weight: 18.5 ⩽ BMI < 25.0, overweight: 25.0 ⩽ BMI < 30, obese: BMI ⩾ 30).

*Eating Disorders in Youth-Questionnaire (EDY-Q)*: The 14-item EDY-Q (van Dyck and Hilbert, 2016) includes one item assessing PB, asking if one likes to eat things that are not meant for eating, and another item measuring RB, asking if one likes to regurgitate food one has already swallowed. In accordance with research in youth (Hartmann *et al*., 2018; Murray *et al*., 2018), while not empirically derived, we defined having engaged in any PB or RB with an item score of ⩾1. Recurrent PB or RB was identified with an item score ⩾4 (i.e. the behaviour was present at least often). Of the other 12 EDY-Q items, ten measure symptoms of ARFID, of which one item was used with altered wording to fit the use in adults (see Hilbert *et al*. (2021) for more details), and two items represent criteria assessing weight and shape concerns. Items are rated on a seven-point Likert scale (0 = *never*; 6 = *always*). Internal consistency of the EDY-Q total mean score (i.e. symptoms of ARFID; without the two items for exclusion criteria and the two measuring PB and RB) amounted to Cronbach's *α* = 0.67 and McDonald's *ω* = 0.51 (Hilbert *et al*., 2021), reflecting the heterogeneity of EDY-Q items.

*Eating Disorder Examination-Questionnaire (EDE-Q)*: The EDE-Q8 (Kliem *et al*., 2016) is a self-report short version of the 28-item EDE-Q (Fairburn and Beglin, 1994; Hilbert and Tuschen-Caffier, 2016) measuring eating disorder psychopathology. Its eight items measure restraint, eating concern, weight concern and shape concern, with two items each, rated on a seven-point scale (0 = *characteristic was not present*; 6 = *characteristic was present every day/in extreme form*) referring to the past 28 days. The mean global score showed excellent internal consistency (Cronbach's *α* = 0.93). Two additional EDE-Q items were used to assess the number of objective binge-eating episodes and compensatory behaviours (aggregated assessment of self-induced vomiting, laxative misuse and driven exercising).

*Questionnaire on the Perception of One's Own Body-9 (FBeK-9)*: The FBeK-9 (Schmalbach *et al*., 2021) is the short version of the German-language FBeK (Strauß and Richter-Appelt, 1996) and consists of nine items assessing subjective aspects of body experience. Items are rated on a five-point scale (0 = *I completely disagree*; 4 = *I completely agree*) and measure attractiveness/self-confidence, accentuation of body/sensitivity and uncertainty/impaired sensation, with three items each. In the present study, only the subscale attractiveness/self-confidence was used to approximate body image. This subscale has shown acceptable reliability across representative and clinical samples (*ω* = 0.53–0.83).

*Patient Health Questionnaire-4 (PHQ-4)*: The four-item PHQ-4 (Löwe *et al*., 2010) is the short form of the Patient Health Questionnaire (Spitzer, 1999 screening for general psychopathology. All items are rated on a four-point Likert scale (0 = *not at all*; 3 = *nearly every day*). Two items each are summed up to form scale scores representing depressive and anxiety symptoms, with higher values indicating greater psychopathology. Both scales show acceptable to good internal consistency (Cronbach's *α* = 0.78 or 0.82).

### Data analysis

Descriptive statistics (frequencies, means and standard deviations, and CI) are used to describe demographic and anthropometric characteristics of participants as well as prevalences of PB and RB. Differences by gender, weight status and education in the groups of individuals with recurrent PB and RB were computed using binary logistic regression models. In the case of a significant prediction of recurrent PB or RB by weight status, another model, controlling for age, gender and socio-economic status was computed. Differences in age, BMI, symptoms of ARFID, eating disorder psychopathology, body image, depression and anxiety between groups with and without recurrent PB and RB, respectively, were analysed using linear regression analyses with recurrent PB or RB, respectively, as the predictor variable. Again, in the case of a significant prediction of the level of BMI by either recurrent behaviour, regression analysis was repeated controlling for age, gender and socio-economic status. Prediction of the occurrence of any binge eating episodes and compensatory behaviours, respectively, by the presence of recurrent PB or RB was tested using binary logistic regression models.

## Results

### Sample characteristics

A total of *n* = 1288 (53.60%) participants identified as women. The overall sample had a mean age of 49.49 ± 17.44 years (range from 18 to 94 years), and 1857 (77.29%) participants reported a duration of education of less than 12 years. Most participants were German (*n* = 2324; 96.71%). The mean BMI of the sample was 25.89 ± 4.68 kg/m^2^ (range from 11.47 to 67.76 kg/m^2^), with *n* = 24 (1.00%) individuals being assigned to the underweight group, *n* = 1108 (46.11%) to the normal-weight group, *n* = 892 (37.12%) to the overweight group and *n* = 339 (14.11%) to the obese group.

### Prevalence of pica and rumination behaviours

Distributions of PB and RB in the total sample (*N* = 2403) are shown in Table 1. Mean scores on both items were low (PB: *M* = 0.13, s.d. = 0.12; RB: *M* = 0.13, s.d. = 0.65). A total of 128 participants (5.33%; 95% CI 4.45–6.20) indicated having experienced PB and 132 (5.49%; 95% CI 4.62–6.37) RB at least once (scores ⩾1 on EDY-Q items). Recurrent PB was reported by 26 participants (1.08%; 95% CI 0.71–1.54) and recurrent RB by 17 participants (0.71%; 95% CI 0.42–1.08) (scores ⩾4 on EDY-Q items).

**Table 1.** Frequency of pica and rumination behaviour in the EDY-Q

Rating	Pica behaviour		Rumination behaviour	
	*n*	%	*n*	%
0 – never true	2275	94.67	2271	94.51
1	54	2.25	55	2.29
2	31	1.29	32	1.33
3	17	0.71	28	1.17
4	9	0.37	6	0.25
5	3	0.12	5	0.21
6 – always true	14	0.58	6	0.25

*Note*: Total *N* = 2403. EDY-Q, Eating Disorders in Youth-Questionnaire.

Recurrent PB and RB co-occurred in six participants (0.25%; 95% CI 0.08–0.45). This corresponds to six of 26 participants with recurrent PB (23.08%; 95% CI 8.33–41.37) and six of 17 participants with recurrent RB (35.29%; 95% CI 13.33–61.11). The frequencies of the two behaviours showed a large correlation (*r* = 0.54, *p* < 0.001).

### Sociodemographic variations of recurrent PB and RB

Regression models did not indicate differences in prevalences of recurrent PB and RB by gender, weight status or education (see Table 2). Of note, the underweight group needed to be excluded from the analyses due to zero cell values. Thus, in contrast to the planned analyses, we built two groups: individuals with normal weight and overweight/obesity. Non-existing group difference in weight status groups therefore refers to this two-group comparison. All omnibus tests indicated that the addition of the variables did not increase the fit of the models (all *p* regarding recurrent PB ⩾0.42; all *p* regarding recurrent RB ⩾0.24).

**Table 2.** Sociodemographic variations of prevalence of recurrent PB and RB

	Recurrent PB						Recurrent RB						
				95% CI for odds ratio						95% CI for odds ratio		
	*B*	s.e.	*p*	LL	OR	UL	*B*	s.e.	*p*	LL	OR	UL
Gender												
Male (ref.)				ref	ref	ref				ref	ref	ref
Female	−0.30	0.40	0.45	0.34	0.74	1.61	−0.26	0.49	0.59	0.30	0.77	2.00
Weight status^a^												
Normal weight (ref.)				ref	ref	ref				ref	ref	ref
Underweight	–	–	–	–	–	–	–	–	–	–	–	–
Overweight/obese	0.59	0.44	0.17	0.77	1.81	4.25	0.50	0.51	0.31	0.61	1.66	4.49
Education												
<12 years (ref.)				ref	ref	ref				ref	ref	ref
⩾12 years	1.26	0.74	0.09	0.07	0.28	1.21	−0.78	0.75	0.30	0.10	0.46	2.00

*Note*: Results from binary logistic regression models on differences in prevalences of recurrent PB and RB, respectively, according to gender, weight status and educational level. Of note, the underweight group needed to be excluded from the analyses due to zero cell values. Thus, in contrast to the planned analyses, we built two groups: individuals with normal weight and overweight/obesity. Total *N* = 2403. PB, pica behaviour; RB, rumination behaviour; CI, confidence interval; OR, odds ratio; LL, lower level; UL, upper level; ref., reference group. ^a^The underweight group needed to be excluded from the analyses due to zero cell values. We have built two groups: individuals with normal weight and individuals with overweight/obesity.

Individuals with recurrent PB or RB, respectively, did not differ from those without recurrent behaviours regarding age (Tables 3 and 4). Results from exploratory analyses of the whole sample (*N* = 2403) by age groups in 10-year brackets are depicted in Table 5. Descriptive findings indicate stable prevalences of recurrent PC across the age groups with the exception of the youngest age group, in which the confidence interval indicated the behaviours to be almost absent. Descriptive findings on RB suggested a stable prevalence only from 40 to 59 years of age, while the CI including zero suggests the data are consistent with a near absence of recurrent RB from 18 to 39 and over 60 years of age.

**Table 3.** Demographic and anthropometric characteristics, eating disorder pathology, symptoms of ARFID, anxiety and depression in individuals with and without recurrent PB

	Recurrent PB median (IQR) (*n* = 26)	No recurrent PB median (IQR) (*n* = 2377)	*b*(s.e.)^a^	*T*	*p*
Age	52.00 (30.00)	50.00 (28.00)	0.01 (3.44)	0.64	0.53
BMI (kg/m^2^)	25.48 (4.43)	25.25 (4.91)	0.02 (0.96)	1.17	0.24
Symptoms of ARFID (EDY-Q)	1.95 (1.45)	0.80 (0.90)	0.16 (0.14)	7.89	<0.01
Eating disorder pathology (EDE-Q8)	1.43 (3.06)	0.25 (1.25)	0.05 (0.25)	2.50	0.01
Attractiveness/self-confidence (FBeK)	19.50 (6.00)	22.00 (5.00)	−0.06 (0.71)	−3.06	<0.01
Depressive symptoms (PHQ-4)	1.00 (2.00)	0.00 (1.00)	0.83 (0.21)	4.08	<0.01
Anxiety symptoms (PHQ-4)	1.00 (3.00)	0.00 (1.00)	0.60 (0.22)	2.92	<0.01

*Note*: Results from linear regression analyses examining differences in variables by groups with and without recurrent PB. IQR, interquartile range; PB, pica behaviour; BMI, body mass index; ARFID, avoidant/restrictive food intake disorder; EDY-Q, Eating Disorders in Youth-Questionnaire; EDE-Q8, Eating Disorder Examination-Questionnaire8; EDE-Q, Eating Disorder Examination-Questionnaire; FBeK, Questionnaire on the Perception of One's Own Body-9; PHQ-4, Patient Health Questionnaire-4.

a Reported are standardised *β* scores.

**Table 4.** Demographic and anthropometric characteristics, eating disorder pathology, symptoms of ARFID, anxiety and depression in individuals with recurrent RB

	Recurrent RB median (IQR) (*n* = 17)	No recurrent RB median (IQR) (*n* = 2386)	*b* (s.e.)^a^	*T*	*p*
Age	49.00 (17.00)	50.00 (28.00)	<−0.01 (4.25)	−0.34	0.73
BMI (kg/m^2^)	25.96 (5.96)	25.25 (4.91)	0.03 (1.14)	1.23	0.22
Symptoms of ARFID (EDY-Q)	3.00 (1.81)	0.80 (0,90)	0.22 (0.17)	10.80	<0.01
Eating disorder pathology (EDE-Q8)	2.13 (1.45)	0.25 (1.25)	0.10 (0.31)	5.15	<0.01
Attractiveness/self-confidence (FBeK)	16.00 (13.00)	22.00 (5.00)	−0.10 (0.89)	−5.13	<0.01
Depressive symptoms (PHQ-4)	2.00 (3.00)	0.00 (1.00)	0.13 (0.28)	6.38	<0.01
Anxiety symptoms (PHQ-4)	2.00 (3.00)	0.00 (1.00)	0.14 (0.28)	6.71	<0.01

*Note:* Results from linear regression analyses examining differences in variables by groups with and without recurrent rumination behaviour. RB, rumination behaviour; IQR, interquartile range; BMI, body mass index; ARFID, avoidant/restrictive food intake disorder; EDY-Q, Eating Disorders in Youth-Questionnaire; EDE-Q8, Eating Disorder Examination-Questionnaire8; EDE-Q, Eating Disorder Examination-Questionnaire; FBeK, Questionnaire on the Perception of One's Own Body-9; PHQ-4, Patient Health Questionnaire-4.

a Reported are standardised *β* scores.

**Table 5.** Exploratory analyses of prevalence of recurrent PB and RB by age groups in 10-year brackets (*N* = 2403)

Age group	Recurrent PB				Recurrent RB			
	95% CI				95% CI			
	*n*	%	LL	UL	*n*	%	LL	UL
18–29 years (*n* = 389)	2	0.51	0.00	1.34	2	0.51	0.00	1.42
30–39 years (*n* = 394)	5	1.27	0.25	2.54	2	0.51	0.00	1.30
40–49 years (*n* = 394)	4	1.02	0.24	2.14	5	1.27	0.26	2.49
50–59 years (*n* = 490)	8	1.63	0.61	3.01	5	1.02	0.21	2.05
⩾60 years (*n* = 736)	7	0.95	0.29	1.74	3	0.41	0.00	0.94

*Note*: PB, pica behaviour; RB, rumination behaviour; CI, confidence interval; LL, lower limit; UL, upper limit.

### Variations of recurrent PB and RB regarding clinical variables

Furthermore, individuals with recurrent PB did not differ from those without recurrent PB in BMI. However, those with recurrent PB reported greater eating disorder psychopathology, more symptoms of ARFID, depression and anxiety, and less positive body image (see Table 3). Group differences between individuals with and without recurrent RB were similar to those between individuals with and without recurrent PB (see Table 4).

In terms of specific eating disorder symptoms, compared to those without, individuals with recurrent PB or RB, respectively, more often reported the occurrence of binge eating episodes (PB: *b*(s.e.) = 1.62 (0.43), *p* < 0.001, OR = 5.08, 95% CI 2.17–11.85; RB: *b*(s.e.) = 1.67 (0.52), *p* < 0.01, OR  =  5.29, 95% CI 1.91–14.69). Compensatory behaviours were only more often reported by individuals with recurrent RB but not by those with recurrent PB compared to their counterparts without recurrent behaviours (PB: *b*(s.e.) = 0.96 (1.04), *p* = 0.35, OR = 2.61, 95% CI 0.34–19.92; RB: *b*(s.e.) = 3.08 (0.61), *p* < 0.001, OR = 21.73, 95% CI 6.61–71.48).

## Discussion

This representative population-based investigation provided the unique opportunity to assess prevalences of one-time and recurrent PB and RB in German adults. Given the placement of both disorders in the category Feeding and Eating Disorders in the DSM-5 (APA, 2013), our second aim was to examine associations with other eating disorder pathology and body image. Finally, we intended to assess the clinical significance of both conditions by measuring comorbid general psychopathology.

We found that 5.33 and 5.49% of adults, respectively, reported having consumed pica substances or performed RB at least once. As expected, recurrent PB and RB were much less frequent (1.08 and 0.71%). In the presence of one recurrent behaviour, the prevalence of the respective other behaviour was significantly higher (35.29% recurrent PB in RB and 23.08% vice versa), and the two were strongly correlated. In contrast, the prevalence of recurrent PB or RB did not differ by gender, weight status or education. Lastly, while individuals with *v*. without recurrent PB and/or RB did not differ in age or BMI, individuals with recurrent PB or RB reported higher eating disorder psychopathology and behavioural symptoms of eating disorders (more compensatory behaviours in RB only), more symptoms of ARFID, anxiety and depression, and less positive body image.

In line with our hypothesis, both recurrent PB and RB were reported less frequently in adults than in youth (recurrent PB: from 3.8 to 5% and recurrent RB: from 1.5 to 1.7% in Hartmann *et al*., 2018 and Murray *et al*., 2018), which might reflect a predominant onset in younger years and/or spontaneous remissions over adolescence. Nevertheless, recurrent PB and RB seem to be clinically significant in adults, as they occur with a similar prevalence to anorexia nervosa (1.4% for women and 0.2% for men; Galmiche *et al*., 2019). While there was no age difference in samples with and without PB or RB, exploratory analyses of age groups by 10-year brackets indicated a relative stability of recurrent PB, but suggested slightly less stability of recurrent RB. However, for both recurrent behaviours, prevalences dropped in early adulthood (from 18 to 29 years). This might represent an age at which shame prevents individuals from reporting this behaviour. Notably, the prevalence of the behaviours did not differ between genders, which is in contrast to findings in youth, in whom at least PB is more common in boys than in girls (Hartmann *et al*., 2018). However, this inconsistency corresponds to the greater prevalences of emotional disorders in boys before puberty and similar rates or a reversal of prevalence between genders during puberty (Wesselhoeft *et al*., 2015). In contrast to previous research (Fawcett *et al*., 2016) that only included pregnant individuals, participants in the present study with different levels of education did not differ in their prevalence of recurrent PB. An exploratory examination of RB indicated no differences in prevalences by education. Lastly, weight status was not associated with the prevalence of either behaviour, which is in line with previous research in youth (Hartmann *et al*., 2018).

Given the inclusion of pica and rumination disorder in the diagnostic category Feeding and Eating Disorders in the DSM-5 (APA, 2013), we were interested in analysing the association of PB and RB with eating disorder pathology. We found greater overall eating disorder psychopathology and specific behavioural symptoms such as objective binge eating in recurrent PB and RB, and more compensatory behaviours in recurrent RB, compared to individuals who did not show these behaviours. These findings partially support the notion that PB and RB might to some extent represent weight control strategies, as suggested by a previous case study in an adult patient with RB (Thomas and Murray, 2016) and by a study of patients in a residential treatment centre for eating disorders (Delaney *et al*., 2015). However, it also cannot be ruled out that the data simply reflect high comorbidities between eating disorder symptoms or epiphenomena of other comorbid disorders. Notably, according to the classification criteria in the DSM-5, if non-nutritive substances are ingested to suppress appetite in the context of anorexia, a pica diagnosis is not warranted, and a current diagnosis of any eating disorder trumps the diagnosis of rumination disorder (APA, 2013). In relation to this, the present study assessed body image, i.e. attractiveness/self-confidence, for the first time in the examination of PB and RB and found associations with both behaviours. Although we measured PB and RB and not the full-threshold disorders, altogether, the results support the inclusion of these disorders in the category Feeding and Eating Disorders (APA, 2013).

Beyond general eating disorder symptoms, the present study is also the first to shed light on associations of recurrent PB and RB with symptoms of ARFID in adults. Consistent with studies in youth (Hartmann *et al*., 2018; Murray *et al*., 2018), adults with recurrent PB or RB reported greater symptoms of ARFID than those without. Interestingly, group differences regarding symptoms of ARFID were larger than for other eating disorder symptoms as reported above, suggesting that ARFID may be more closely linked to PB and RB. It should be noted, however, that symptoms of ARFID, PB and RB were assessed with the same instrument, potentially leading to an overestimation of their association.

In terms of associated mental burden, in contrast to studies in youth (Hartmann *et al*., 2018) but in line with our hypothesis for adults, individuals with both recurrent PB and RB showed greater symptoms of depression and anxiety than those without these recurrent behaviours. Compared to youth, adults might be more aware of the fact that the behaviours are not normative, might feel ashamed, fear medical consequences or might be more strongly impacted by the behaviours. Alternatively, the behaviours might also merely be an epiphenomenon of the other disorders. A study by Liu *et al*. (2021), e.g. highlighted that pica might be related to a complex comorbidity structure, insofar as it is strongly associated with restless legs syndrome, which, in turn, is associated with depressive symptoms (e.g. Ulfberg *et al*., 2007). Further studies are needed to elucidate these associations.

The present findings need to be interpreted against the background of some strengths and limitations. Strengths include the representative sampling and large sample size. However, it needs to be acknowledged that our prevalences of overweight and obesity were significantly lower than in the large representative ‘German Health Interview and Examination Survey for Adults’ (DEGS1 study, *N* = 7116) conducted by the Robert Koch Institute between 2008 and 2011 (Mensink *et al*., 2013) using objectively measured body weight and height (overweight: 37.1% in our study *v*. 53% in women and 61.3% in men; obesity: 14.1% in our study *v*. 23.3 and 23.9%, respectively). This may partially be due to the fact that our participants self-reported their height and weight. Additionally, our sample comprised slightly more female participants compared to the data of the Federal Statistical Office from 2018 (53.6 *v*. 50.7%). Finally, compared to data from the Federal Statistical Office for 2020, we had slightly more participants from younger age groups (<60 years: 69.4 *v*. 63.0%). Thus, the generalisability of our findings across the German adult population is slightly limited. A further strength lies in the assessment of other general and eating disorder psychopathology using validated instruments. Limitations include the one-item assessment of PB and RB, which was used due to the lack of diagnostic instruments and does not allow for a precise diagnostic categorisation. Moreover, the EDY-Q measure only has anchors for both ends of the seven-point Likert scale (with 0 = ‘never’ to 6 = ‘always’). Furthermore, while the items are worded in the present tense, they do not clearly indicate the time point for which participants are asked to report behaviours. Thus, the kind of prevalence reported (lifetime *v*. period *v*. point) cannot be pinpointed. When interpreting the findings, particularly the group differences, it needs to be acknowledged that despite the large number of participants, the power of the study was limited due to the low prevalences. Additionally, the survey did not include tests measuring intellectual disabilities, which have proven to be a frequent comorbid presentation in individuals of various age groups (Ali, 2001) and might have differential diagnostic value. However, given our participants' ability to read, understand and answer our survey, our sample presumably included barely any participants with, if at all, a minor intellectual disability. Moreover, we were not able to assess medical diseases that might account for the disorders or eating disorders that trump a rumination diagnosis. These aspects need to be kept in mind when interpreting prevalences of both behaviours reported here. As a diagnosis of pica can only be inferred if the substance is non-food and non-nutritive, and particularly as a small descriptive survey in a convenience sample of adolescents and adults indicated that half of the pica substances mentioned would not qualify as such (Hartmann, 2019), future studies should also assess the kind of substance(s) consumed. On the other hand, individuals might not have reported certain items they consume (e.g. paper or freezer frost) due to how the question was formulated. Thus, the lack of inquiry into the substances consumed might have led to either an under- or overestimation of PB. Finally, as the survey was completed with research assistants present, the findings may not be fully comparable to studies using self-administered surveys.

This study has both clinical and research implications. For clinicians, the study uniquely highlights that PB and RB exist in adults. Given the documented association with eating disorder and general psychopathology and the potential detrimental medical consequences of PB and RB, they should be assessed during admissions for eating disorder treatment in both outpatient and residential settings, and in other settings where individuals with eating disorders including ARFID present (e.g. gastroenterological services). In view of the increased comorbid presentation of the two behaviours, the same holds true when patients report one of the behaviours. With regard to research implications, further population-based studies are needed to replicate the present findings, and German-language diagnostic instruments comparable to the English Pica, ARFID and Rumination Disorder Interview (PARDI; Bryant-Waugh *et al*., 2019) need to be developed and validated to allow for a diagnosis of full-syndrome pica and rumination disorder. To shed light on the need for treatment, it is necessary to examine the prevalence and severity of medical consequences and the natural course of the behaviours and disorders.

In summary, this is the first study to provide a population-based estimate of the prevalence of PB and RB in adults. While prevalences of recurrent PB and RB were low (<1 %), they are comparable to those of anorexia nervosa and should not be overlooked. The clinical significance of the behaviours is further highlighted by their potential detrimental health consequences, and associations with greater symptoms of eating disorders, including ARFID, as well as depression and anxiety.

## Data

Data pertaining to this study are available upon reasonable request from the last author (AH).
